# Intravascular haemolysis and acute kidney injury following atrial fibrillation ablation: a report using two different systems for pulsed field ablation

**DOI:** 10.1093/europace/euae251

**Published:** 2024-10-01

**Authors:** Maarten A J De Smet, Clara François, Benjamin De Becker, Rene Tavernier, Jean-Benoît le Polain de Waroux, Sébastien Knecht, Mattias Duytschaever

**Affiliations:** Department of Cardiology, AZ Sint-Jan Hospital Bruges, Ruddershove 10, B-8000 Brugge, Belgium; Department of Cardiology, AZ Sint-Jan Hospital Bruges, Ruddershove 10, B-8000 Brugge, Belgium; Department of Cardiology, AZ Sint-Jan Hospital Bruges, Ruddershove 10, B-8000 Brugge, Belgium; Department of Cardiology, AZ Sint-Jan Hospital Bruges, Ruddershove 10, B-8000 Brugge, Belgium; Department of Cardiology, AZ Sint-Jan Hospital Bruges, Ruddershove 10, B-8000 Brugge, Belgium; Department of Cardiology, AZ Sint-Jan Hospital Bruges, Ruddershove 10, B-8000 Brugge, Belgium; Department of Cardiology, AZ Sint-Jan Hospital Bruges, Ruddershove 10, B-8000 Brugge, Belgium

**Keywords:** Atrial fibrillation, Ablation, Pulsed field, Radiofrequency, Acute kidney injury, Haemolysis

Pulmonary vein isolation (PVI) is an established cornerstone of rhythm management in patients with atrial fibrillation (AF).^1,2^ Before pulsed field ablation (PFA), PVI lesions were solely created by thermal radiofrequency (RF), cryo, or laser energy. Pulsed field ablation, resulting in irreversible electroporation of cell membranes to which cardiac muscle is especially sensible, is the first non-thermal energy that provides effective PVI with good durability, including non-inferiority when compared with RF.^2,3^

Initial reports showed an excellent clinical safety profile for PFA.^3,4^ More recently, two cases of severe acute kidney injury (AKI) secondary to intravascular haemolysis (IH) have been described following AF ablation with 174 and 126 PF applications.^5^ Also, another study reported increased serum creatinine and haemoglobinuria following a higher number of PF applications without hydration.^6^ In MANIFEST-17k, the largest worldwide PFA registry, 5 out of more than 17 000 patients (0.03%) were reported to have transient haemolysis-related kidney failure requiring temporary dialysis (143 ± 27 PF applications).^7^ All these reports only studied the pentaspline PFA catheter since its clinical approval in 2021 and agreed on the fact that some degree of haemolysis occurs in most patients and is proportionate to the number of PF applications.^5,7,8^ So far, no study compared novel PFA technologies on the market and the question remains whether this effect also occurs in these technologies.

Here, we assessed peri-procedural IH and kidney function following PVI-only or PVI with additional lesion sets (PVI-plus group; roof and posterior mitral isthmus lines, vein of Marshall ethanolization, and/or posterior wall isolation) using two commercially available PFA systems (Varipulse variable loop PFA catheter, Biosense Webster; Farawave pentaspline PFA catheter, Boston Scientific) or RF energy (ThermoCool SmartTouch, Biosense Webster). Variable loop PFA procedures used the CARTO 3 system (Biosense Webster Inc., Diamond Bar, CA, USA) for mapping. For the pentaspline PFA catheter, PV angiograms were performed unless contra-indicated [contrast allergy, risk of thyrotoxicosis, advanced chronic kidney disease (CKD)]. Patients provided a blood sample before ablation and a blood and urine sample 24 h post-ablation. Acute kidney injury was classified according to the Kidney Disease Improving Global Outcomes classification: serum creatinine 1.5–1.9 times baseline or ≥0.3 mg/dL increase (Stage 1), 2–2.9 times baseline (Stage 2), and 3 times baseline or increase of serum creatinine ≥ 4 mg/dL or initiation of renal replacement therapy (RRT) (Stage 3). The study was carried out according to the principles of the Declaration of Helsinki and was approved by the local ethical committee. Data are reported as mean ± standard deviation or median (IQR). Comparative statistics were performed using one-way ANOVA or Student’s *t*-test for normally distributed continuous variables and *χ*^2^ test for percentages or categorical data. The Mann–Whitney test was used for non-uniformly distributed data.

One hundred ninety-eight consecutive patients underwent a first ablation procedure using the variable loop PFA catheter in 54 out of 198 patients (27%), the pentaspline PFA catheter in 98 patients (49%), or RF in 46 patients (24%). Pulmonary vein isolation-only ablation was performed in 129 out of 198 patients (65%). In 69 patients (35%), PVI-plus was performed. Demographic and clinical characteristics were not significantly different between ablation modalities (*Table 1*). However, the PVI-plus group showed significantly more CKD, structural heart disease (SHD) and heart failure, lower LVEF, and larger left atria as compared with PVI-only. Mean RF applications were 68 ± 15 (range 38–106) for PVI-only and 88 ± 15 (range 57–118) for PVI-plus with a mean total RF ablation (RFA) time of 1336 ± 445 s and 1866 ± 565 s, respectively. Using the variable loop PFA catheter, mean applications were 46 ± 6 (range 36–64) for PVI-only and 62 ± 11 (range 50–83) for PVI-plus, whereas these were 33 ± 2 (range 32–44) and 46 ± 6 (range 32–66) applications, respectively, for the pentaspline catheter.

**Table 1:** Summary table showing demographics, clinical and procedural characteristics, and haemolysis parameters following radiofrequency ablation, variable loop PFA, and pentaspline PFA

Baseline characteristics	Radiofrequency		Variable loop PFA		Pentaspline PFA	
	PVI-only (*n* = 32)	PVI-plus (*n* = 14)	PVI-only (*n* = 40)	PVI-plus (*n* = 14)	PVI-only (*n* = 57)	PVI-plus (*n* = 41)
Age (years)	68 ± 10	71 ± 8	66 ± 11	67 ± 9	65 ± 12	69 ± 8
Female, *n* (%)	14 (44)	4 (29)	12 (30)	5 (36)	24 (42)	18 (44)
SHD, *n* (%)	12 (38)	8 (57)*	10 (25)	6 (43)*	17 (30)	14 (34)
CAD, *n*	6	4	5	1	8	8
DCM, *n*	1	0	0	1	0	0
HCM, *n*	1	1	1	0	2	0
VHD, *n*	2	0	1	1	1	0
Congenital, *n*	1	0	0	0	2	0
TCMP, *n*	1	3	3	0	4	6
Heart failure, *n* (%)	6 (19)	9 (64)*	10 (25)	6 (43)*	12 (21)	12 (29)
HFpEF, *n*	2	3	4	2	2	3
HFmrEF, *n*	3	5	4	2	7	6
HFrEF, *n*	1	1	2	2	3	3
LVEF (%)	61 ± 7	55 ± 11*	62 ± 5	55 ± 17*	63 ± 8	58 ± 11**
LAD (mm)	41 ± 5	45 ± 4**	39 ± 5	47 ± 8***	39 ± 6	43 ± 6***
LAVI (mL/m²)	27 ± 10	37 ± 14*	29 ± 8	37 ± 11*	29 ± 9	45 ± 10***
CKD, *n* (%)	3 (9)	5 (36)**	1 (3)	5 (36)**	8 (14)	11 (27)*
Serum creatinine (mg/dL)	1.01 ± 0.34	1.03 ± 0.28	0.91 ± 0.16	0.99 ± 0.2	0.94 ± 0.4	0.99 ± 0.3
eGFR (CKD-EPI, mL/min)	73 ± 20	71 ± 18	83 ± 14	75 ± 17	80 ± 18	70 ± 18**
DM, *n* (%)	4 (13)	6 (43)*	3 (8)	2 (14)	7 (12)	7 (17)
AHT, *n* (%)	15 (47)	8 (57)	10 (25)	8 (57)	21 (37)	22 (54)
Previous stroke, *n* (%)	1 (3)	1 (7)	3 (8)	0 (0)	8 (14)	5 (12)
PAD, *n* (%)	0 (0)	0 (0)	1 (3)	0 (0)	1 (2)	3 (7)
CHA_2_DS_2_-Vasc score	2 (1–4)	3 (2–5)	2 (0–3)	3 (1–4)	2 (1–3)	3 (2–5)*

Outcomes	Radiofrequency	Variable loop PFA		Pentaspline PFA		
	No AKI (*n* = 46)	No AKI (*n* = 50)	Stage 1 (*n* = 4)	No AKI (*n* = 94)	Stage 1 (*n* = 3)	Stage 3 (*n* = 1)
Age (years)	69 ± 9	63 ± 13	69 ± 6	68 ± 10	72 ± 7	78
Female, *n* (%)	21 (46)	16 (32)	1 (25)	42 (45)	0 (0)	0 (0)
PVI-plus, *n* (%)	14 (30)	12 (24)	2 (50)	38 (40)	3 (100)	0 (0)
CKD, *n* (%)	17 (37)	6 (12)	3 (75)	27 (29)	2 (75)	1 (100)
DM, *n* (%)	10 (22)	3 (6)	2 (50)	13 (14)	1 (33)	1 (100)
AHT, *n* (%)	23 (50)	16 (32)	3 (75)	40 (43)	2 (75)	1 (100)
SHD, *n* (%)	20 (43)	6 (12)	2 (50)	30 (32)	2 (75)	1 (100)
Heart failure, *n* (%)	15 (33)	14 (28)	2 (50)	21 (22)	2 (75)	1 (100)
LVEF (%)	59 ± 9	62 ± 8	43 ± 22	61 ± 10	42 ± 17	40
CHA_2_DS_2_-Vasc score	2 (2–4)	2 (0–3)	3 (3–3)	2 (1–4)	5 (4–5)	6
RF time (s) or PF applications	1501 ± 540	50 ± 10	58 ± 16	38 ± 7	50 ± 11	32
Change in serum creatinine (mg/dL)	−0.1 ± 0.11	−0.08 ± 0.07	0.38 ± 0.06	−0.07 ± 0.07	0.37 ± 0.1	0.21
Free plasma Hb (mg/L)	189 ± 181	335 ± 211	943 ± 1106	477 ± 330	756 ± 201	1600
Haptoglobin (g/L)	1.1 ± 0.49	0.58 ± 0.36	0.02 ± 0.01	0.82 ± 0.51	0.44 ± 0.16	0.2
Total bilirubin (mg/dL)	0.83 ± 0.38	1.4 ± 0.65	2.75 ± 0.9	1.07 ± 0.6	2.04 ± 1.54	3.9
LDH (U/L)	211 ± 36	215 ± 37	322 ± 75	261 ± 51	362 ± 83	403
Haemoglobinuria, *n* (%)	3 (7)	5 (10)	4 (100)	6 (6)	3 (100)	1 (100)

Data are means ± standard deviation or median (IQR). *, **, and *** indicate *P* < 0.05, *P* < 0.01, or *P* < 0.001, respectively, as compared with PVI-only. For clarity, only statistically significant *P*-values are indicated.

AHT, arterial hypertension; AKI, acute kidney injury; CAD, coronary artery disease; CKD, chronic kidney disease; DCM, dilated cardiomyopathy; DM, diabetes mellitus; eGFR, estimated glomerular filtration rate; Hb, haemoglobin; HCM, hypertrophic cardiomyopathy; HFmrEF, heart failure with mid-range ejection fraction; HFpEF, heart failure with preserved ejection fraction; HFrEF, heart failure with reduced ejection fraction; LDH, lactate dehydrogenase; LAD, left atrial diameter; LAVI, left atrial volume index; LVEF, left ventricular ejection fraction; PAD, peripheral artery disease; PVI, pulmonary vein isolation; SHD, structural heart disease; TCMP, tachycardiomyopathy; VHD, vascular heart disease.

Regardless of ablation modality, total haemoglobin was significantly (*P* < 0.0001) reduced post-ablation without significant differences in the PVI-only and PVI-plus groups (*Figure 1A*). No patient required transfusion. However, haptoglobin was significantly decreased after PFA (*P* < 0.0001) and not following RFA. Twenty-four hours after ablation with either PF modality, this was most pronounced for PVI-plus as compared with PVI-only (*P* < 0.0001). Additionally, reticulocyte count, free plasma haemoglobin, total bilirubin, and lactate dehydrogenase significantly increased post-ablation for the variable loop and pentaspline PFA catheters (*P* < 0.0001), but not for RF. This was more so in the PVI-plus group as compared with the PVI-only group (*P*-values ranging from 0.0242 to 0.0031 for variable loop and all *P* < 0.0001 for pentaspline PFA). Twenty-four hours after PFA, a significant proportion of patients showed haemoglobinuria (3%, 15%, and 11% following RF, variable loop, or pentaspline PVI-only ablation, respectively, and 7%, 29%, and 33% for PVI-plus ablation) (*Figure 1A*). No significant differences in IH were observed between the two PFA catheters.

**Figure 1:**
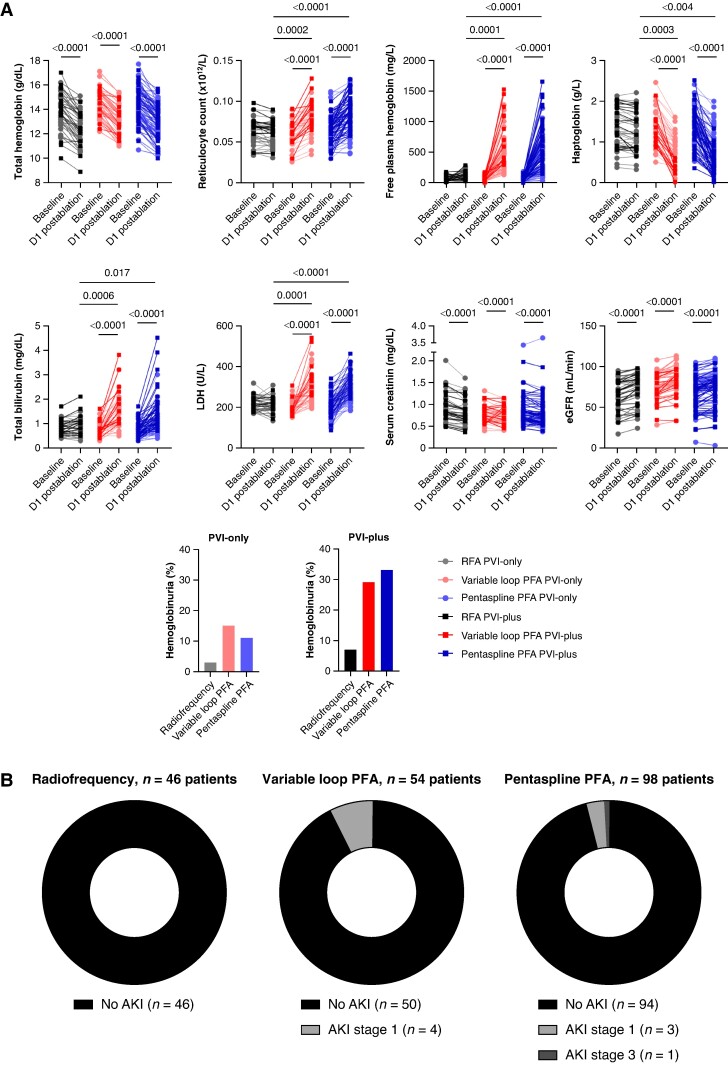
(*A*) Pre- and 24 h post-ablation blood sample and urine analysis for RFA (*n* = 46 patients), variable loop PFA (*n* = 54 patients), and pentaspline PFA (*n* = 98 patients). For each graph: RFA is indicated on the left (black), variable loop PFA in the middle (red) and pentaspline PFA on the right (blue). PVI-only is indicated more transparently, while PVI-plus is indicated in full color. For clarity, only statistically significant *P*-values are shown on the graphs. (*B*) Parts of whole graphs indicating patients with and without AKI following RFA, variable loop PFA, and pentaspline PFA. AKI, acute kidney injury; eGFR, estimated glomerular filtration rate; LDH, lactate dehydrogenase; PFA, pulsed field ablation; RFA, radiofrequency ablation.

Globally, serum creatinine decreased and estimated glomerular filtration rate (eGFR) improved post-ablation in all groups (*P* < 0.0001). However, using PFA, eight patients developed AKI (four Stage 1 following variable loop PFA, three Stage 1, and one Stage 3 following pentaspline PFA; *Figure 1B*). No AKI was observed following RFA (*P* = 0.04 and 0.007 vs. variable loop and pentaspline PFA, respectively). No significant differences in renal outcomes were observed between the variable loop and pentaspline PFA catheter (*P* = 0.26). Acute kidney injury occurred more in older patients, with known CKD, diabetes mellitus, and arterial hypertension, with SHD and more depressed LVEF and with a higher number of PF applications and more pronounced haemolysis (*Table 1*). Stage 3 AKI occurred in one patient with known advanced CKD (baseline eGFR 14 mL/min) following PVI-only (32 PF applications, no pulmonary vein angiograms) pentaspline PFA. Following ablation, he developed progressive renal injury (serum creatinine from 3.58 to 3.79 mg/dL) with hyperkalaemia and was initiated on RRT. All patients recovered back to baseline kidney function, except for the patient with Stage 3 AKI.

Our study confirms (i) that peri-procedural haemolysis occurs in a significant proportion of patients following PFA, (ii) that it is more pronounced when PVI is associated with additional lesion sets, and (iii) that it confers a risk of AKI.^5,7,8^ We add that this effect occurs regardless of the type of PFA catheter used. This effect was not observed following RFA and contrasts a recent publication by Osmancik *et al*.^9^ where borderline changes in haemolysis parameters were also observed following RFA. In the same publication, 1 out of 47 patients treated with PFA showed AKI Stage 1 (2.13%), which is in line with our reported 5.26% (8 out of 152 PFA cases). We add that AKI patients are generally older, more likely received PVI-plus ablation, and have CKD, heart failure, diabetes mellitus, and arterial hypertension. Our findings corroborate that prognosis is good, except for advanced CKD. However, where previous studies emphasized a larger number of PF applications necessary for renal injury, our data suggest that even a standard number of applications may provide deleterious effects. As such, additional factors such as tissue contact and the possible effect of PV angiograms on post-procedural kidney function may be essential in these patients.^10^

In conclusion, PFA-associated renal injury exists with both the pentaspline and variable loop PFA catheter. In the setting of advanced CKD, progression of kidney failure may occur, also with a limited number of PF applications.

## Data Availability

The data and analysis methods that support the findings of this study are available from the corresponding author upon reasonable request.
